# Draft Genome Sequence of the Bacterium *Pseudomonas putida* CBB5, Which Can Utilize Caffeine as a Sole Carbon and Nitrogen Source

**DOI:** 10.1128/genomeA.00640-15

**Published:** 2015-06-11

**Authors:** Erik M. Quandt, Ryan M. Summers, Mani V. Subramanian, Jeffrey E. Barrick

**Affiliations:** a Center for Systems and Synthetic Biology, Center for Computational Biology and Bioinformatics, Institute for Cellular and Molecular Biology, Department of Molecular Biosciences, The University of Texas at Austin, Austin, Texas, USA; b Department of Chemical and Biological Engineering, The University of Alabama, Tuscaloosa, Alabama, USA; c Center for Biocatalysis and Bioprocessing, Department of Chemical and Biochemical Engineering, The University of Iowa, Iowa City, Iowa, USA

## Abstract

*Pseudomonas putida* CBB5 was isolated from soil by enriching for growth on caffeine (1,3,7-trimethylxanthine). The draft genome of this strain is 6.9 Mb, with 5,941 predicted coding sequences. It includes the previously studied Alx gene cluster encoding alkylxanthine *N*-demethylase enzymes and other genes that enable the degradation of purine alkaloids.

## GENOME ANNOUNCEMENT

Due to its widespread use as a stimulant in coffee and other beverages, caffeine is often found in the environment, particularly in the pulp remaining after extracting beans from coffee berries (1) and in surface waters around human population centers (2). Related purine alkaloids have uses in medicine, including as bronchodilators for treating asthma. Interest in developing enzymatic routes for caffeine demethylation for drug production and bioremediation has led to the discovery of several microbes that can break down caffeine (3, 4). *Pseudomonas putida* CBB5 was isolated from a soil sample in Coralville, IA, by enriching for microbes capable of utilizing caffeine as a sole carbon and nitrogen source (5). It was found to degrade caffeine and other purine alkaloids by a sequential *N*-demethylation pathway (6, 7).

The *N*-demethylation activity of *P. putida* CBB5 was localized to an ~14-kb cloned DNA fragment covering most of an alkylxanthine (Alx) degradation gene cluster. The Alx gene cluster contains multiple Rieske nonheme iron monooxygenases that are involved in these enzymatic steps (6). Five of the Alx genes have been shown to be sufficient for caffeine degradation to xanthine by refactoring them into an operon that functions in *Escherichia coli* (8). We sequenced the genome of CBB5 to gain a more complete understanding of other genetic determinants in this strain that underlie its ability to utilize caffeine and other alkylxanthines.

We assembled a draft genome for CBB5 using a combination of paired-end and mate pair libraries sequenced on an Illumina HiSeq instrument. The A5 pipeline (version 20140604) (9, 10) was used for *de novo* assembly of reads after the removal of adaptor sequences using FlexBar (version 2.31) (11). The resulting assembly was screened for contaminating sequences and annotated using the NCBI Prokaryotic Genome Annotation Pipeline. The final draft genome consists of 146 contigs, with an *N*_50_ of 213,667 bp. It has a total size of 6.9 Mb and 60.0% G+C content. The 16S rRNA gene of CBB5 is 99% identical to the 16S sequences of *Pseudomonas fluorescens* Pf0-1 (12) and *Pseudomonas* sp. UW4 (13).

We annotated the Alx gene cluster to reflect the characterization of the five *N*-demethylase genes (*ndmABCDE*), two putative alkylxanthine-responsive transcriptional regulators (*cafT* and *cafR*), and a putative caffeine permease (*cafP*). Nine base pair differences from previously published sequences were found in this region (6, 7). Genome-wide, we found eight putative glutathione-dependent formaldehyde dehydrogenase genes (similar to *E. coli* *frmA* or *frmB*) that may allow CBB5 to assimilate formaldehyde generated via *N*-demethylation (7). CBB5 is also known to contain a second pathway that can degrade methylxanthines to methyl uronic acids (5). We identified three operons with putative xanthine dehydrogenase genes (similar to *E. coli* *xdhABC*) that are candidates for encoding this function. The genome sequence of CBB5 will be useful for further understanding how it utilizes various alkylxanthines as carbon and nitrogen sources.

### Nucleotide sequence accession numbers.

This whole-genome shotgun project has been deposited in DDBJ/EMBL/GenBank under the accession no. JTEN00000000. The version described in this paper is version JTEN01000000.
